# Growth of Tellurium Nanobelts on h-BN for *p*-type Transistors with Ultrahigh Hole Mobility

**DOI:** 10.1007/s40820-022-00852-2

**Published:** 2022-04-19

**Authors:** Peng Yang, Jiajia Zha, Guoyun Gao, Long Zheng, Haoxin Huang, Yunpeng Xia, Songcen Xu, Tengfei Xiong, Zhuomin Zhang, Zhengbao Yang, Ye Chen, Dong-Keun Ki, Juin J. Liou, Wugang Liao, Chaoliang Tan

**Affiliations:** 1 College of Electronics and Information Engineering, Shenzhen University, Shenzhen, 518060 People’s Republic of China; 2 Department of Electrical Engineering, City University of Hong Kong, Hong Kong SAR, People’s Republic of China; 3 Department of Physics, The University of Hong Kong, Pokfulam Road, Hong Kong SAR, People’s Republic of China; 4 Department of Chemistry, The Chinese University of Hong Kong, Hong Kong SAR, People’s Republic of China; 5 Department of Chemistry, City University of Hong Kong, Hong Kong SAR, People’s Republic of China; 6 Department of Mechanical Engineering, City University of Hong Kong, Hong Kong SAR, People’s Republic of China; 7 Center of Super-Diamond and Advanced Films (COSDAF), City University of Hong Kong, Hong Kong SAR, People’s Republic of China

**Keywords:** Chemical vapor deposition, Substrate engineering, Tellurium, Field-effect transistors, Hole mobility

## Abstract

The lack of stable *p*-type van der Waals (vdW) semiconductors with high hole mobility severely impedes the step of low-dimensional materials entering the industrial circle. Although *p*-type black phosphorus (bP) and tellurium (Te) have shown promising hole mobilities, the instability under ambient conditions of bP and relatively low hole mobility of Te remain as daunting issues. Here we report the growth of high-quality Te nanobelts on atomically flat hexagonal boron nitride (h-BN) for high-performance *p*-type field-effect transistors (FETs). Importantly, the Te-based FET exhibits an ultrahigh hole mobility up to 1370 cm^2^ V^−1^ s^−1^ at room temperature, that may lay the foundation for the future high-performance *p*-type 2D FET and metal–oxide–semiconductor (p-MOS) inverter. The vdW h-BN dielectric substrate not only provides an ultra-flat surface without dangling bonds for growth of high-quality Te nanobelts, but also reduces the scattering centers at the interface between the channel material and the dielectric layer, thus resulting in the ultrahigh hole mobility .

**Supplementary Information:**

The online version contains supplementary material available at 10.1007/s40820-022-00852-2.

## Highlights

- The growth of high-quality single-crystalline Te nanobelts is reported by introducing atomically flat hexagonal boron nitride (h-BN) nanoflakes into the chemical vapor deposition system as the growth substrate.
- The field-effect transistor based on Te grown on h-BN exhibits an ultrahigh hole mobility up to 1370 cm^2^ V^−1^ s^−1^ at room temperature.

**Supplementary Information:**

The online version contains supplementary material available at 10.1007/s40820-022-00852-2.

## Introduction

Van der Waals (vdW) materials hold great promise in fabricating advanced monolithic integrated circuits by virtue of the absence of the dangling bonds and their superior electrical properties [1–10]. As the basic unit in the monolithic integrated circuits, the complementary metaloxide–semiconductor (CMOS) is one of the most important architectures. In which, *n*-type field-effective transistors (FETs) and *p*-type FETs are both indispensable. Although multiple *n*-type FETs have been demonstrated based on various *n*-type 2D semiconductors [11–16], the development of their *p*-type counterparts is still an urgent need. Since Li et al. first reported the black phosphorus (bP)-based FETs in 2014, the bP once became an alternative option for researchers to prepare *p*-type FETs [17–20]. However, bP shows poor stability under ambient conditions and thus it has not received as much attention as its *n*-type counterparts for instance MoS_2_ [21, 22]. The situation has not improved until the rediscovery of tellurium (Te) by Wang et al. [23], their *p*-type FETs based on the Te nanoflakes by hydrothermal synthesis showed a considerable hole mobility of 700 cm^2^ V^−1^ s^−1^ [23]. Meanwhile, their fabricated Te FETs showed a great air stability without any encapsulation. Moreover, superior mechanical and thermoelectric properties of Te endow it with great potential in flexible electronics and wearable sensors [24, 25]. However, considering the electrical transport property of Te is dominated by several factors including structural defects, phonon scattering, charge impurities, surface traps, and the used organic solvents in the hydrothermal method may increase trap densities, it is believed that there is still plenty of room by using other growth strategy to obtain high-quality single-crystalline Te nanostructures with higher hole mobilities.

Substrate engineering is well accepted as an effective growth strategy to obtain high-quality single-crystalline vdW materials [26–31]. For example, by introducing the liquid gold substrate in the chemical vapor deposition (CVD) system, the ultrathin wafer-scale single-crystalline hexagonal boron nitride (h-BN) film can be grown via self-collimated grain formation [27]. Except for h-BN, large-scale single-crystalline MoS_2_ can also be grown on Au (111) substrate in a CVD system [28]. In a recent study, 2 inch single-crystalline monolayer MoS_2_ thin films has been synthesized on the sapphire substrate, and the observed electron mobility was 102.6 cm^2^ V^−1^ s^−1^ [26]. Moreover, the epitaxial growth of single-domain graphene on h-BN with a fixed stacking orientation has been reported [30]. And in another work, an extremely high Hall mobility was observed in graphene synthesized via CVD on h-BN [31]. Apart from CVD method, the substrate engineering can also be introduced into molecular beam epitaxy (MBE) system, for example, by using which, large-scale single-crystalline MoSe_2_ monolayer has been grown on h-BN substrate [29].

In this work, we report a promising strategy for growth of high-quality single-crystalline Te nanobelts with high hole mobilities by introducing atomically flat h-BN nanoflakes into the CVD system as the growth substrate. The single-crystalline nature of the synthesized Te nanobelts is characterized by the high-resolution transmission electron microscope (HRTEM). In addition, our synthesized Te nanobelts exhibit an obvious optical anisotropy as characterized by Raman spectroscopy. Importantly, the FET based on the synthesized Te nanobelts presents a typical *p*-type transfer characteristic with a field-effect hole mobility up to 1370 cm^2^ V^−1^ s^−1^ at room temperature, which is much higher than the previously reported value in FETs based on Te nanoflakes synthesized by the hydrothermal method and other CVD methods [23, 32, 33].

## Experimental Section

### Growth of Te Nanobelts

The Te nanobelts were grown on h-BN nanoflakes mechanically exfoliated onto the SiO_2_/Si wafer. The thickness of the SiO_2_ dielectric layer is 300 nm. The bulk h-BN crystals were purchased from 2D Semiconductor Inc. A quartz boat containing 50 mg TeO_2_ powder (99.999%, Aladdin) was placed at the center of the heating zone of the tube furnace, and the SiO_2_/Si substrate caped with discrete h-BN nanoflakes was put at the downstream end of the tube outside the heating zone. Before reaction, the tube was pumped to 1 $\times$ 10^−2^ Torr to sweep away the air and then refilled by Ar/H_2_ hybrid gas (the volume ratio of H_2_ is 10%) to atmospheric pressure. During the reaction, a constant mixture of Ar (90 sccm) and H_2_ (10 sccm) was used as the carrier gas and served as reductive agent at the same time. The pressure of the tube was kept as atmospheric pressure. The temperature of the furnace ramped up to 750–800 °C in 40 min, and was maintained at the peak temperature for 20 min. The crystallization temperature for Te nanobelts was 170–200 °C which can be controlled by adjusting the distance of the SiO_2_/Si wafer from the heating zone. After the reaction, the furnace was cooled down naturally to room temperature.

### Device Fabrication

The Te FETs based on the h-BN/SiO_2_/Si and bare SiO_2_/Si substrates in global bottom-gate geometry were prepared by using the electron-beam lithography (EBL) technique without transfer. The electrodes were defined by EBL (TESCAN, VEGA3). gold (Au) (70 nm) was used as contact metal for source/drain electrodes, and achieving a good ohmic contact between the metal and Te nanobelts. On the other hand, the fabrication of Te FETs in local bottom-gate geometry started from the pre-pattern of local bottom-gate electrode, which was defined by EBL and followed by the deposition of chromium (Cr) (5 nm) and Au (60 nm). Then the Te nanobelts together with h-BN substrates were transferred onto the bottom electrode via a wet-transfer method. Similarly, Au (70 nm) was used as contact metal for source/drain electrodes, and a good ohmic contact was realized.

### Material Characterization

The morphologies of Te nanobelts were characterized by optical microscopy (Nikon, ECLIPSE LV100ND) and scanning electron microscopy (SEM) (TESCAN, VEGA3). The surface morphologies and heights of the synthesized Te nanobelts were measured by atomic force microscope (AFM) (Bruker, Dimension Icon with Scan Asyst). The Raman spectrum and mapping were measured by Renishaw with a polarized incident laser at room temperature, the wavelength of the excitation laser is 532 nm. The Raman peak of a silicon wafer at 520 cm^−1^ was used as the reference to calibrate the spectrometer. High-resolution transmission electron microscope (HRTEM) images were obtained by Tecnai F20 TEM (TF20).

### Electrical Characterization

The room-temperature electrical measurements were performed in a cryogenic probe station (LakeShore) in vacuum with a source meter (Keysight B2902B). The low-temperature electrical measurements were conducted in a home-built cryostat with a dry closed cycle cryocoolers (Sumltomo Heavy Industries, Ltd.) under 5 × 10^−6^ Torr with Keithley’s standard series 2400-c source meters controlled by LabVIEW program. Before the measurements, the fabricated Te FET was connected to pins of a ceramic package by indium wires via wire bonding.

## Results and Discussion

### Growth of Te Nanobelts

**Fig. 1:**
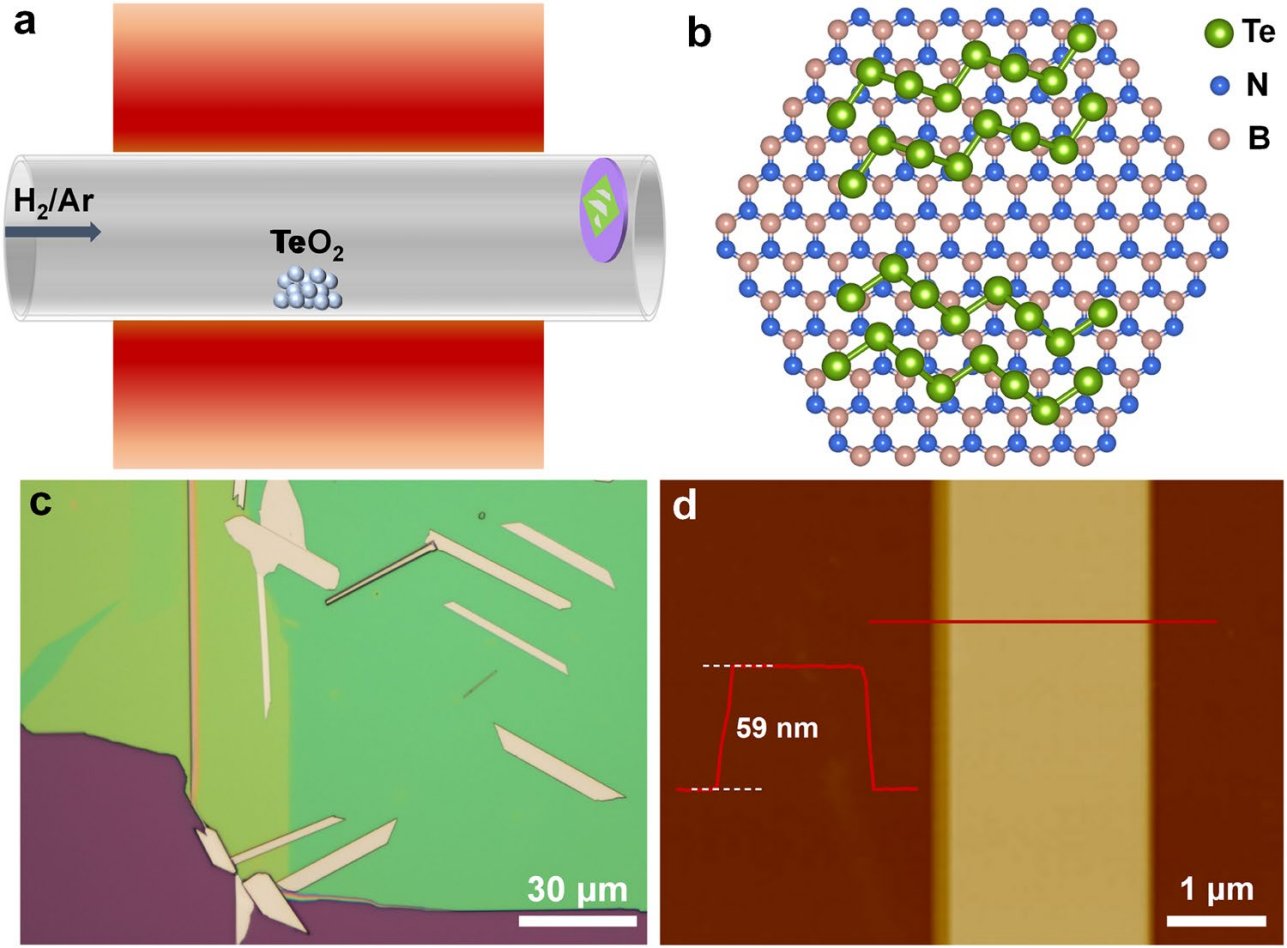
**a** Schematic illustration of the growth of Te nanobelts. **b** Schematic illustration of Te crystal structure on h-BN crystal structure in a top view. **c** Typical optical image of Te nanobelts grown on h-BN. **d** AFM image of a typical Te nanobelt with thickness of 59 nm, the inset shows the height profile corresponding to the red line across the sample

Single-crystalline Te is composed of individual helical chains stacked via vdW force interactions and within each molecular chain, one Te atom is strongly covalently-bonded with two neighboring atoms along the long-axis [23, 34–36]. The schematic illustration of the Te nanobelts growth is shown in Fig. 1a, where the tellurium dioxide (TeO_2_) powder was used as the precursor material and the hydrogen/argon (H_2_/Ar) hybrid gas helped reduce TeO_2_ and served as carrier gas. In the growth process, after completing the placement of the sample and growth substrate under the ambient conditions, the quartz tube was first vacuumed and then inflated to atmospheric pressure by H_2_/Ar hybrid gas. When the precursor was heated up to ~750 °C, TeO_2_ was reduced by the H_2_ and then the Te atoms moved to the growth zone assisted by the Ar carrier gas. In the growth zone, we put the silicon (Si) wafer caped by 300 nm silicon dioxide (SiO_2_) as the substrate, on which distributed the h-BN nanoflakes obtained by the mechanical exfoliation. After the reaction, single-crystalline Te nanobelts with length of tens of micrometers (μm) and width of several micrometers were found on the substrate. Figure 1b shows the scheme of the crystal structure of the Te nanobelts on the h-BN substrate in a top view. The *c*-axis of our Te crystals is along the surface of the h-BN nanoflakes, which is different from the ultrathin Te nanoflakes grown by other CVD method and physical vapor deposition (PVD) process, in which, the molecular chains in the obtained Te nanoflakes are perpendicular to the substrate surface [37, 38]. The introduction of h-BN nanoflakes in our growth strategy can provide an atomically flat surface for the growth of Te nanobelts and reduce the probable surface trap states, leading to the synthesis of high-quality Te nanobelts. As shown in Figs. 1c and S1b, which are the optical images of typical Te nanobelts on h-BN nanoflakes and bare SiO_2_/Si wafer, respectively, most of samples are in the rectangular and trapezoid shapes. The length and width of the Te nanobelts can reach up to 50 and 10 μm, respectively. The morphologies of Te samples are quite similar to the solution-synthesized Te nanoflakes [23, 32]. The typical thickness of the synthesized Te nanobelts ranges from 30 to 70 nm, which is shown in Figs. 1d and S1c–d. We note that the measured height profiles of these samples were extremely uniform, which indicates that there are no organic residues on the surface of Te nanobelts, unlike those synthesized by hydrothermal method [32]. Besides Te nanobelts, Te nanowires could also be synthesized by adjusting the distance of the substrate from the heating zone to control the substrate temperature, the obtained samples are shown in Fig. S1a, this growth phenomenon can be explained by the different surface energies of Te crystals as reported before [33]. The nucleation energy along the *c*-axis is the lowest, which means the highest growth rate along the *c*-axis (that is of [001] orientation). In the low-temperature region, the growth rate is much higher compared with growth rates of other orientations, thus leading to Te nanowires. In high-temperature regions, the growth energy becomes higher, the growth rates of [100] and [10 $\overline{1 }$] orientations are comparable to the growth rate along the *c*-axis, so that Te nanobelts in rectangular and trapezoid shapes also appear. The temperature-dependent behavior of the growth of Te crystals provides us the opportunity to control the morphologies of Te crystals in our growth strategy in future.

### Raman and TEM Characterizations of Te Nanobelts

**Fig. 2:**
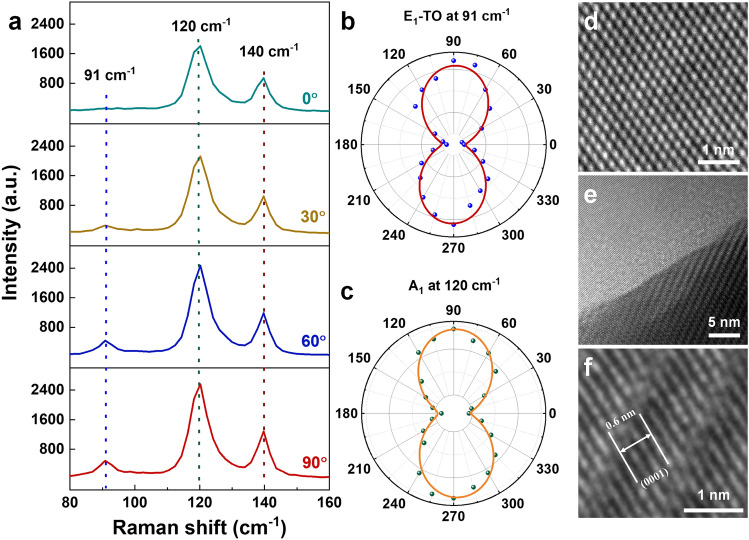
**a** Angle-resolved Raman spectra of a Te nanobelt with thickness of 30 nm with angles between the crystal orientation and incident laser polarization. **b, c** Polar figures of Raman intensity corresponding to *E*_1_-TO mode located at 91 cm^−1^ (**b**) and *A*_1_ mode located at 120 cm^−1^ (**c**). **d–f** HRTEM images of Te nanobelts grown on h-BN; **d** bare h-BN, **e** edge of Te nanobelt on h-BN, **f** a zoomed-in area of Te nanobelt grown on h-BN from **e**

The structure anisotropy of a typical synthesized Te nanobelt with the thickness of ~30 nm is further analyzed by angle-resolved Raman spectra at room temperature. Figure 2a shows the Raman spectra with angles between the crystal orientation and the polarization of the incident laser. From which, we can see that there are three Raman peaks locate at 91 cm^−1^ (*E*_1_ transverse (TO) phonon mode), 120 cm^−1^ (*A*_1_ mode) and 140 cm^−1^ (*E*_2_ mode), respectively, and the absence of the longitudinal (LO) phonon mode is consistent with the Raman features of thick Te nanostructures reported elsewhere [23, 32]. By rotating the Te nanobelt in steps of 15°, we observed nearly no changes in the peak locations; however, the change of the peak intensities is obvious. We extracted the peak intensities of *E*_1_-TO and *A*_1_ modes by fitting with a sine function and plotted them into the polar figures (Fig. 2b–c). It is obvious that the intensities of these Raman peaks present periodic changes with the rotation angles. Moreover, both *E*_1_-TO and *A*_1_ modes show the maximum intensity at 90 and 270°, by considering 0° indicates polarization of the used laser is parallel with the long-axis of the Te nanobelts, that confirms the helical chain is along the long-axis of our samples [23]. The Raman intensity mapping of *A*_1_ mode with the angles of 90 and 0° is shown in Fig. S2. The uniform Raman signal across the whole Te nanobelts demonstrates that the quality of Te nanobelts is quite uniform. To further identify the crystal structure of the Te nanobelts, HRTEM was used to measure the lattice structure of the Te nanobelts on h-BN nanoflakes, the measured results are shown in Fig. 2d–f. Figure 2d is the HRTEM image of the h-BN area, which was mechanically exfoliated onto the SiO_2_/Si substrate. The perfect hexagonal lattice without defects demonstrates the high quality of the used h-BN substrate, which is the precondition for the high-quality Te nanobelts growth. Figure 2e shows the edge of a Te nanobelt grown on h-BN, the well-defined and continuous crystal lattice of the Te nanobelt demonstrates its good quality. The zoomed-in HRTEM image of the Te nanobelt stacked on h-BN in Fig. 2e is shown in Fig. 2f. From which, we can see that the measured lattice constant of Te nanobelt is 0.2 nm, which is assignable to the (0001) lattice direction parallel with the helical chains, in agreement with the Te nanoflakes synthesized by hydrothermal method [23, 32].

### Electrical Performance of Te FET with Global Bottom-gate Structure

**Fig. 3:**
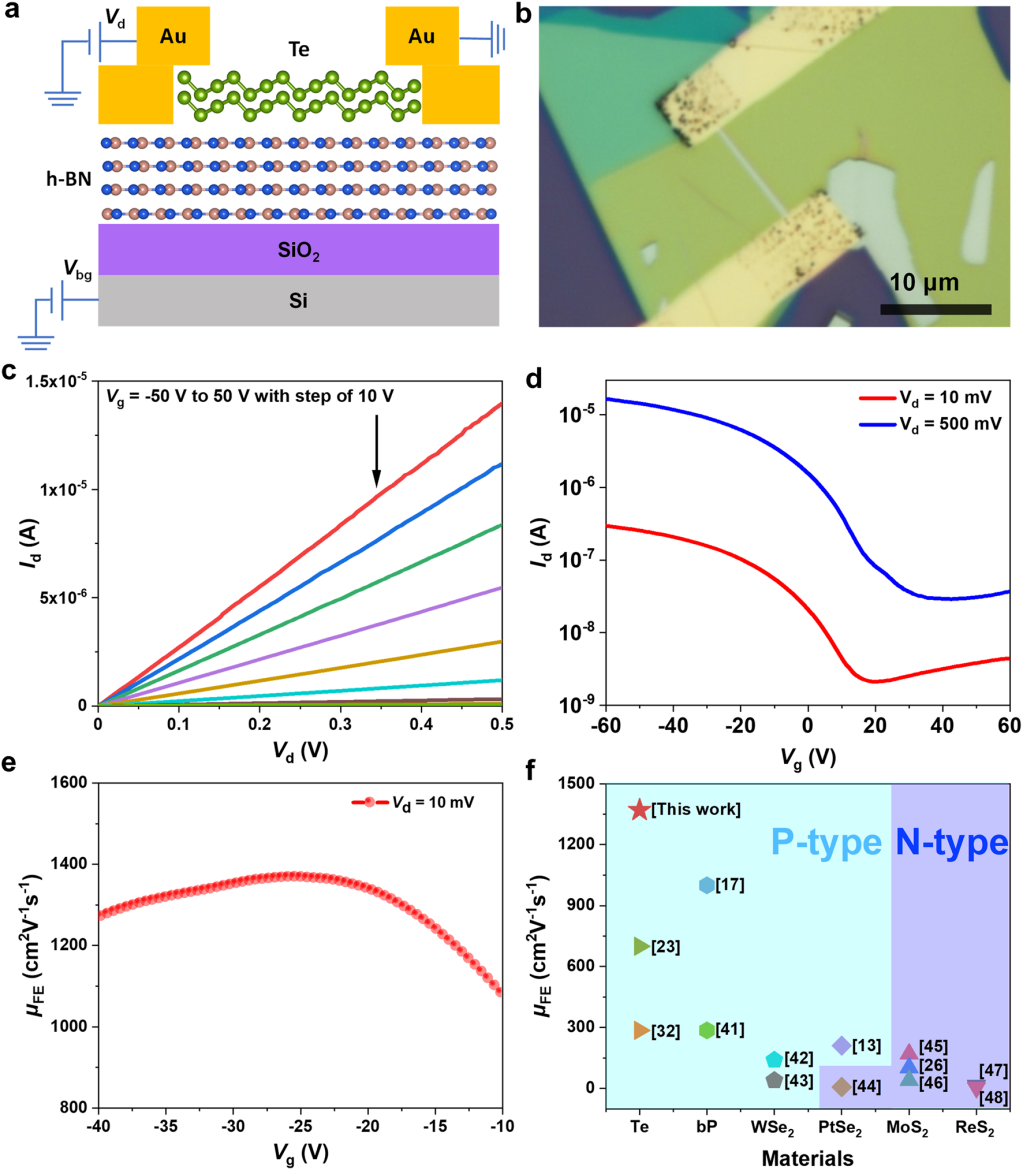
**a** Schematic illustration of Te-based FET with global bottom-gate structure on h-BN/SiO_2_/Si substrate in a cross-sectional view. **b** Optical image of a typical Te FET on h-BN/SiO_2_/Si. **c** Output and **d** transfer curves of Te FET measured at room temperature. **e** Field-effect mobility of Te transistor extracted from the transfer curves under the bias of *V*_d_ = 10 mV in panel *d*. **f** Summary of field-effect mobility of our synthesized Te crystal with other vdW materials with high room-temperature mobilities reported in literature

The vdW h-BN dielectric layer with atomically flat surface provides an ideal platform for the growth of high-quality single-crystalline Te nanobelts. Moreover, the free of dangling bonds on the surface of h-BN nanoflakes leads to low density of charge-scattering centers and charge trap states, which will result in high carrier mobilities observed in the channel material [39, 40]. To investigate the electrical transport properties of the Te nanobelts in our growth strategy, we first fabricated FET with global bottom-gate structure directly on the Te nanobelts grown on h-BN substrate without further transfer, the scheme of the device architecture is shown in Fig. 3a. The heavily doped silicon was used as a global bottom-gate and the source/drain electrodes were patterned by EBL. The thickness of the SiO_2_ layer is 300 nm. To reduce the contact resistance, Au (70 nm) was evaporated by thermal evaporation as the electrodes. Figure 3b is the optical image of a typical device, the thickness of the channel material is 30 nm, which is indicated by Fig. S3b. Figure S3a shows the detailed SEM image of this device. Output and transfer curves of this device are shown in Fig. 3c–d. It can be concluded from Fig. 3c that the ohmic contact has been realized for the source-drain current $\left( {I_{{\text{d}}} } \right)$ changes linearly with the bias voltage $\left( {V_{{\text{d}}} } \right)$ under different back-gate voltages. Figure 3d shows that the transfer curves of our device under the source-drain bias of 10 and 500 mV, respectively. The transfer curves showing in this figure present a *p*-type-dominant slightly ambipolar behavior and that suggests the superior crystal quality of our samples. Limited by the thickness of our Te nanobelt, the on/off ratio only reaches ~10^2^. This relatively low on/off ratio can be explained by the thickness-dependent bandgap of Te, which reduces to ~0.3 eV when the thickness of Te is larger than 20 nm [23, 35]. At the same time, the gate voltage exhibits an inferior control over the thick channel material. The energy band diagram of this device is shown in Fig. S3c–d. When a negative voltage is applied on the bottom gate, holes will be accumulated in the channel, thus increasing its conductivity significantly (“on” state); on the other hand, once a positive voltage is applied on the bottom gate, holes will be depleted, and the conducting channel will be turned off (“off” state).

The field-effect hole mobilities of our Te FETs can be derived by inserting numbers into the following formula:

1 $$\mu_{{{\text{FE}}}} = \frac{{g_{{\text{m}}} L}}{{W \times C_{{\text{g}}} \times V_{{{\text{ds}}}} }}$$

where $g_{{\text{m}}}$, $L$, $W$, and $C_{{\text{g}}}$ are the transconductance, channel length, channel width, and h-BN/SiO_2_ capacitance, respectively. The calculation result under the bias voltage of 10 mV at room temperature is shown in Fig. 3e, in which device the thickness of h-BN/SiO_2_ is 82/300 nm, and we can extract a peak $\mu_{{{\text{FE}}}}$ of 1370 cm^2^ V^−1^ s^−1^. This value is much higher than the room-temperature field-effect hole mobility (984 cm^2^ V^−1^ s^−1^) extracted in bP FET [17]. In Fig. 3f, we summarize the reported room-temperature field-effect mobilities of typical vdW semiconductors including solution-synthesized Te [23, 32], bP [17, 41], tungsten diselenide (WSe_2_) [42, 43], platinum diselenide (PtSe_2_) [13, 44], molybdenum disulfide (MoS_2_) [26, 45, 46], and rhenium disulfide (ReS_2_) [47, 48]. It can be seen that the hole mobility of our device is the highest among the *p*-type vdW semiconductors and compared with the well-known TMD materials, our Te FETs show much higher room-temperature mobility. In addition, our device shows a good air stability. We have remeasured the electrical performance of this device with one-week air exposure and the measured results are shown in Fig. S4. The transfer curve of our device measured after one week is similar to the as-prepared one under the same bias voltage, which is shown in Fig. S4a. The extracted peak hole mobility as shown in Fig. S4b is 1376 cm^2^ V^−1^ s^−1^, which is nearly the same as the value (1370 cm^2^ V^−1^ s^−1^) extracted from the as-prepared device. The much higher stability of Te FET can compensate for the drawback of bP originating from the unstable nature in air conditions.

To further confirm the high hole mobilities observed in the nanobelts synthesized by our growth strategy, we make the statistics on the hole mobilities and on/off ratios of the FETs fabricated on the Te nanobelts grown on h-BN substrates. The calculated results are shown in Table S1. In all these fabricated devices, the hole mobilities range from 847–1370 cm^2^ V^−1^ s^−1^, and the average hole mobility is 1108 cm^2^ V^−1^ s^−1^, which is higher than the reported value in bP FET [17]. Limited by the thickness of our Te samples, the on/off ratios found in our devices are in the range of ~10–10^2^. Considering the on/off ratios in vdW material-based FETs can be improved further by employing dual-gate architectures, it is expected that there is still sufficient room for further improvement on the performances of our FETs [49–52]. As a control group, we also fabricated FETs based on Te nanobelts grown on the SiO_2_/Si wafer but without h-BN nanoflakes in the same CVD method. Its performance is shown in Fig. S5. Figure S5a is the optical image of a typical device. In which, same to the FETs fabricated on Te nanobelts grown on h-BN nanoflakes, the heavily doped silicon was used as the global bottom-gate. The thickness of the used dielectric SiO_2_ layer is 300 nm. The 70 nm Au has been deposited as contact electrodes. The linear output curves shown in Fig. S5b indicate the realization of ohmic contact. Figure S5c shows the transfer curves of the measured device under the bias voltage of 0.1 and 0.5 V, respectively. Its on/off ratio is ~3. The field-effect hole mobility $\mu_{{{\text{FE}}}}$ is extracted from its transfer curve according to Eq. (1), and the result is shown in Fig. S5d. The peak value of its mobility is 375 cm^2^ V^−1^ s^−1^ under the source-drain voltage of 0.1 V. The calculated field-effect hole mobilities and on/off ratios of all the fabricated Te FETs based on bare SiO_2_/Si substrate are listed in Table S2, the average $\mu_{{{\text{FE}}}}$ among these devices is 394 cm^2^ V^−1^ s^−1^, which is similar to the effective mobility extracted in Te nanostructures synthesized by hydrothermal method and other CVD method [32, 33]. This much more inferior mobility also further indicates the superiority of our growth strategy. And the huge improvement on $\mu_{{{\text{FE}}}}$ in FETs fabricated on Te nanobelts grown on h-BN substrate compared with those on bare SiO_2_/Si substrate is believed to originate from the better crystal quality and the reduced carrier scattering induced by the atomically flat surface provided by the introduced h-BN nanoflakes.

To better understand the electrical transport properties of the Te nanobelts synthesized by our growth strategy, we performed the temperature-dependent electrical characterization of one typical Te FET. The optical image of this device is shown in Fig. S6a, the thickness of the channel material is ~ 30 nm. The transfer curves of our device measured over a temperature range from 30 to 300 K are shown in Fig. S6b. It’s obvious that the on/off ratio of the Te FET increases when the device is cooled, this can be ascribed to the small-band-gap nature of Te, that is, as the temperature being elevated, the thermal generation will increase the carrier densities dramatically. An on/off ratio of 420 is obtained at the temperature of 30 K, while when the temperature increases to 300 K, the on/off ratio decreases to 10. The extracted field-effect mobility reduces as temperature increases as well, which is shown in Fig. S6c. Our device shows a $\mu_{{{\text{FE}}}}$ of 3500, 2548, and 1184 cm^2^ V^−1^ s^−1^ at 30, 70, and 300 K, respectively. To show the reliability of the calculated field-effect mobility values, we replotted the temperature dependence of the *I*_ds_–*V*_g_ on linear scale as shown in Fig. S7. Where the reliability factor $r_{{{\text{in}}}}$ of the claimed mobility values extracted in the linear regime at different temperature was obtained by calculating the ratio of the slope of the black dashed line to the slope of the pink dashed line in each panel [53]. The black dashed line corresponds to the mobility of an electrically equivalent FET following the ideal Shockley behavior and has a zero-contact resistance; and the claimed field-effect mobility in the manuscript was extracted from the pink dashed line. Figure S7 clearly shows that as temperature increase, the threshold voltage of the measured device approaches zero in general and $r_{{{\text{in}}}}$ increases from 186% at 30 K to 107% at 300 K, that suggests that under low temperature, the effect of the contact on the device is non-negligible and leads to an overestimation of the mobility values. However, as the temperature increases to room temperature (300 K), our claimed mobility presents a high reliability $\left( {r_{{{\text{in}}}} = 93\% } \right)$. Meanwhile, the observed temperature dependence of field-effect mobility can be fitted with a power law $\mu_{{{\text{FE}}}} \propto T^{ - \gamma }$, in our case $\gamma = 0.48$, this probably indicates the observed mobility in our device is limited by phonon scattering rather than charge impurities [32, 39]. Which further demonstrates that the introduced h-BN substrate provides a dielectric layer with less charge-scattering centers for Te channel. In addition, the band gap of the used Te nanobelt can be estimated via the temperature-dependent minimum drain current $I_{{\text{d,min}}}$, which is determined by thermal activation of carriers over the band gap of the Te nanobelt [32, 54],

2 $$I_{{{\text{d}},\,\min }} \propto \exp \left( { - \,\frac{{E_{{\text{g}}} }}{{k_{{\text{B}}} T}}} \right)$$

where $k_{{\text{B}}}$ is the Boltzmann constant, $E_{{\text{g}}}$ is the transport band gap, and $T$ is the temperature. On the basis of the low-temperature electrical measurement on our device, we extract a band gap of $E_{{\text{g}}} = 0.2{\text{ eV}}$. It is worth pointing out that the band gap extracted for thick samples in this way is usually underestimated and that can be attributed to the measured thick Te sample cannot be effectively turned off by the used gate voltage for gate electric field only depletes channel region within Debye length. In another word, the carriers in Te channel region beyond Debye length remains undepleted and thus lead to a high off-state current in the measurement [32, 55].

### Electrical Performance of Te FET with Local Bottom-gate Structure

**Fig. 4:**
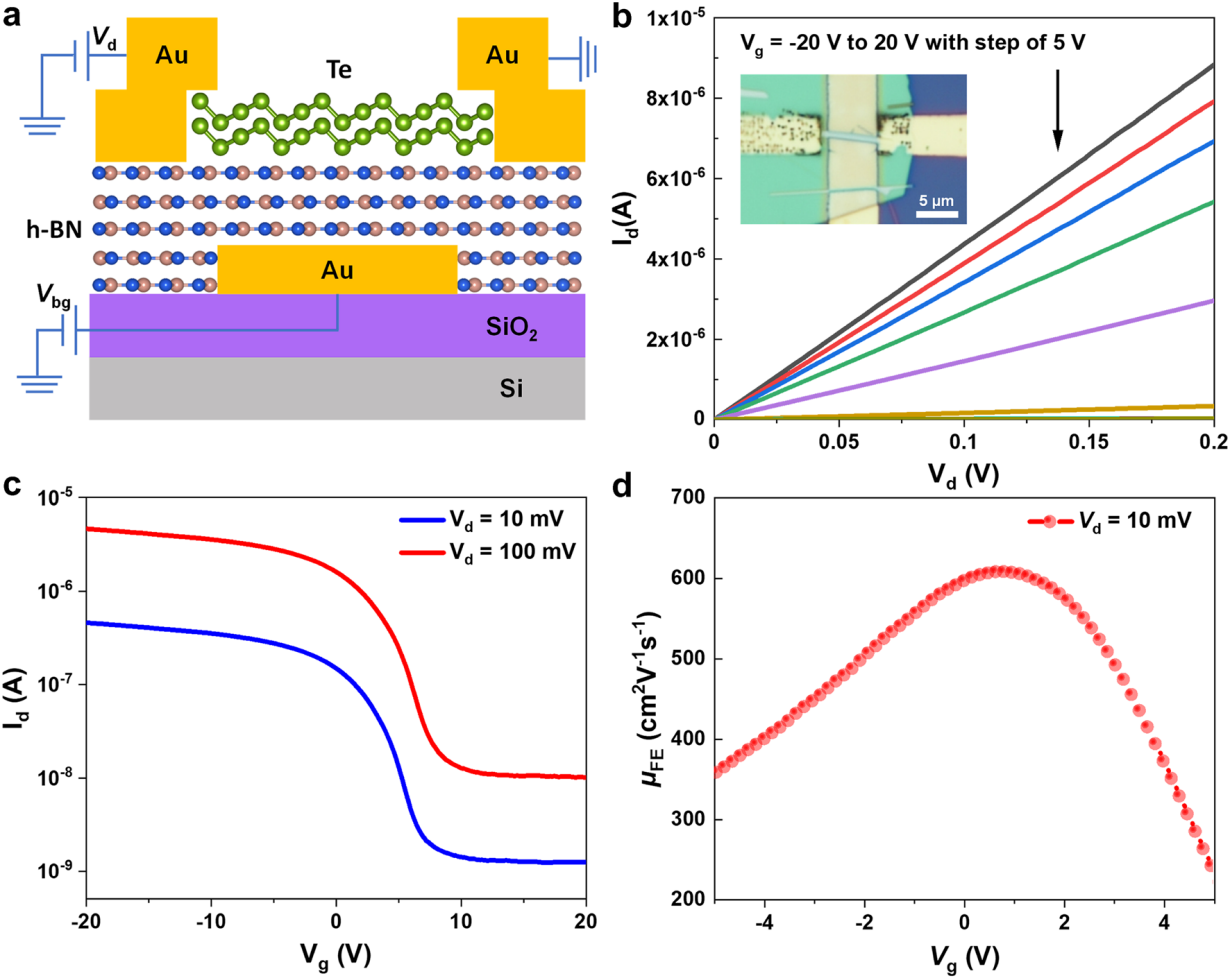
**a** Schematic illustration of local bottom-gate Te FET by using h-BN as dielectric layer in a cross-sectional view. **b** Output curves of a typical Te FET. The inset shows the optical image of the measured Te FET with local bottom-gate structure. **c** Transfer curves of the same Te FET device under different bias measured at room temperature. **d** Field-effect mobility of Te transistor extracted from the transfer curve under the bias of *V*_d_ = 10 mV in panel *c*

In the end, we demonstrated the construction of Te FET in local bottom-gate architecture. Compared with global-gate geometry, the local gate usually exhibits a better control over the channel material and can be applied in building logic gates and circuits [1, 2, 56, 57]. The local bottom-gate was patterned by EBL followed by the deposition of 5 nm Cr and 60 nm Au, respectively. After that, the Te nanobelts with the h-BN nanoflakes beneath were together transferred from the SiO_2_/Si wafer onto the local bottom-gate in a wet-transfer method [58]. In this way, the bottom interface between the Te nanobelt and the h-BN dielectric layer is expected to be protected from contamination. Similarly, the Au with the thickness of 70 nm was deposited as source/drain electrodes. Figure 4a illustrates the scheme of this device and the optical image of one typical device is shown in the inset in Fig. 4b. Figure S8a shows the detailed SEM image of this device, the white dotted rectangular box indicates the location of the channel material. Figure S8b is the AFM image of our device. The thickness of the chosen Te nanobelt is 30 nm. The output curves measured in this device are shown in Fig. 4b, the linear relationship of the source-drain current versus source-drain voltage under different gate voltages indicates the realization of ohmic contact. Figure 4c shows the transfer curves measured at room temperature on this device, compared with Te FET with channel material in equal thickness, this device shows a much larger on/off ratio of 370 and 460 under the bias of 100 and 10 mV, respectively. The larger on/off ratios indicate a better gate control over channel material in local-gate geometry. From the transfer curve under the bias of 10 mV in Fig. 4c, we extracted the field-effect mobility in Fig. 4d. The peak value is $\mu_{{{\text{FE}}}} \, = \,608\;{\text{cm}}^{2} \;{\text{V}}^{ - 1}{\text{s}}^{ - 1}$, which is only half of the calculated value in the global-gate Te FET, this may be explained by the more scattering centers induced by the wet-transfer method. Moreover, future complexed logic gates and even circuits may be designed and constructed on the basis of our demonstration of Te FET in local-gate geometry.

## Conclusion

In summary, we have developed a substrate engineering-based CVD strategy for growth of high-quality single-crystalline Te nanobelts for fabrication of high-performance *p*-type FETs. The introduced h-BN nanoflakes in the CVD system not only provide the growth substrate with atomically flat surface for the synthesis of high-quality Te nanobelts, but also reduce the scattering centers in the subsequent Te FETs, finally leading to a significant improvement on its field-effect hole mobility. The high quality of the synthesized Te nanobelts was confirmed by HRTEM. Compared with the solution-synthesized single-crystalline Te nanoflakes, our samples show a much cleaner surface. In addition, consistent with the previous reports on Te nanostructures, our samples show significant optical anisotropy, which is characterized by Raman spectra. Moreover, the uniform Raman signal in the Raman mapping on our samples also suggests their uniform qualities. We obtained an ultrahigh $\mu_{{{\text{FE}}}}$ of 1370 cm^2^ V^−1^ s^−1^ in our Te nanobelts grown on the h-BN substrates. The much smaller $\mu_{{{\text{FE}}}}$ in Te grown on bare SiO_2_/Si wafer confirms the critical role of h-BN in our growth strategy. In the end, we demonstrated the Te FET in local-gate geometry, which may provide guideline for future Te-based logic gates and circuits. Such high-mobility *p*-type FETs may find their applications in highly integrated vdW semiconductor-based logic circuits, thus promoting the rapid development of vdW semiconductor-based nanoelectronics.

## Supplementary Information

Below is the link to the electronic supplementary material.

Supplementary file1 (PDF 724 KB)
