# Supplementary material for: Growth of Tellurium Nanobelts on h-BN for p-type Transistors with Ultrahigh Hole Mobility
Source: Nanomicro Lett. 2022 Apr 19;14:109. doi: 10.1007/s40820-022-00852-2 (PMC9018950; doi:10.1007/s40820-022-00852-2)
Supplement: Supplementary file 1 — Supplementary file1 (PDF 724 KB) [file 40820_2022_852_MOESM1_ESM.pdf]

Supporting Information for

Growth of Tellurium Nanobelts on h-BN for p-Type Transistors with Ultrahigh Hole Mobility

Peng Yang[1, 2], Jiajia Zha[2, *], Guoyun Gao[3], Long Zheng[4], Haoxin huang[2], Yunpeng Xia[2], Songcen Xu[2], Tengfei Xiong[5], Zhuomin Zhang[6], Zhengbao Yang[6], Ye Chen[4], Dong-Keun Ki[3], Juin J. Liou[1], Wugang Liao[1, *], Chaoliang Tan[2, 7, *]

[1] College of Electronics and Information Engineering, Shenzhen University, Shenzhen 518060, P. R. China

[2] Department of Electrical Engineering, City University of Hong Kong, Hong Kong SAR, P. R. China

[3] Department of Physics, The University of Hong Kong, Pokfulam Road, Hong Kong SAR, P. R. China

[4] Department of Chemistry, The Chinese University of Hong Kong, Hong Kong SAR, P. R. China

[5] Department of Chemistry, City University of Hong Kong, Hong Kong SAR, P. R. China

[6] Department of Mechanical Engineering, City University of Hong Kong, Hong Kong SAR, P. R. China

[7] Center of Super-Diamond and Advanced Films (COSDAF), City University of Hong Kong, Hong Kong SAR, P. R. China

*Corresponding authors. E-mail: jiajiazha2-c@my.cityu.edu.hk (J. Zha); wgliao@szu.edu.cn (W. Liao); chaoltan@cityu.edu.hk (C. Tan)

Supplementary Figures and Tables

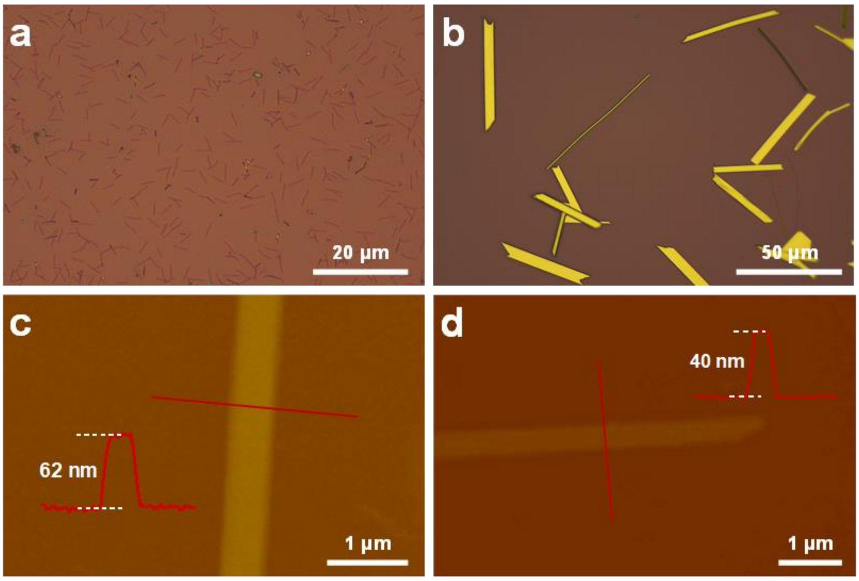

Fig. S1 Optical and AFM characterizations of as-grown Te samples. (**a, b**) Typical optical images of low-dimensional Te crystals grown at different temperatures on SiO_2/Si via CVD method, (**a**) 170 °C, (**b**) 200 °C. (**c, d**) AFM images of Te nanobelts grown on h-BN with thickness of 62 nm (**c**), and 40 nm as indicated by the corresponding inset height profiles

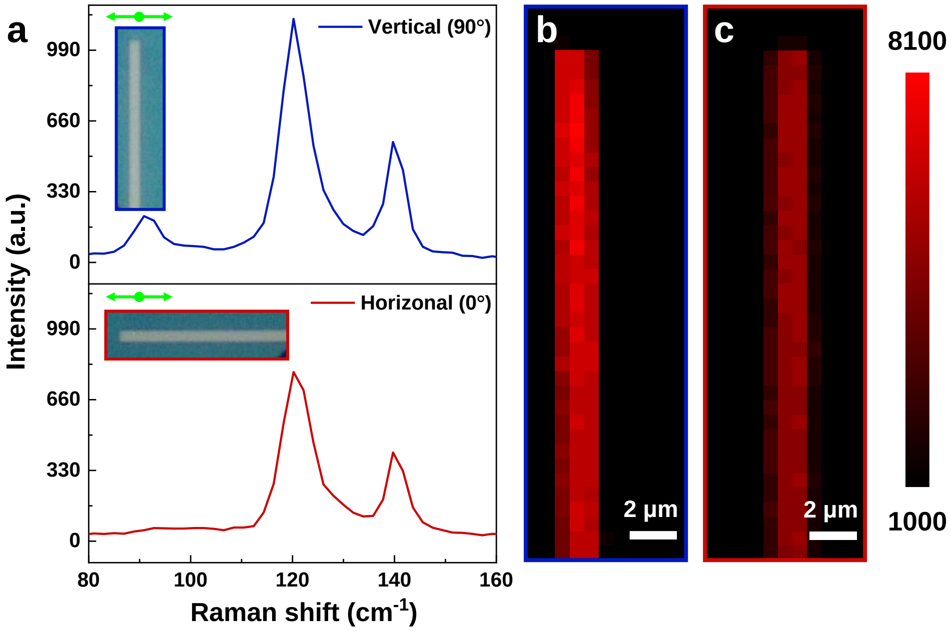

Fig. S2 Angle-resolved Raman characterizations of Te nanobelt. (**a**) Polarization Raman spectra of a Te nanobelt with angles between [001] crystal orientation and incident laser polarization, the angles in upper and lower panel are 90° and 0°, respectively. The insets show the incident laser polarization and the optical images of rotated Te nanobelts. (**b, c**) Polarization peak intensity Raman mapping of A_1 mode located at 120 cm^{-1}, The angles between [001] orientation of Te crystal and incident laser polarization are 90° (b) and 0° (**c**), respectively. The corresponding optical images are shown in the inset of (**a**) surrounded by rectangular boxes with the same color

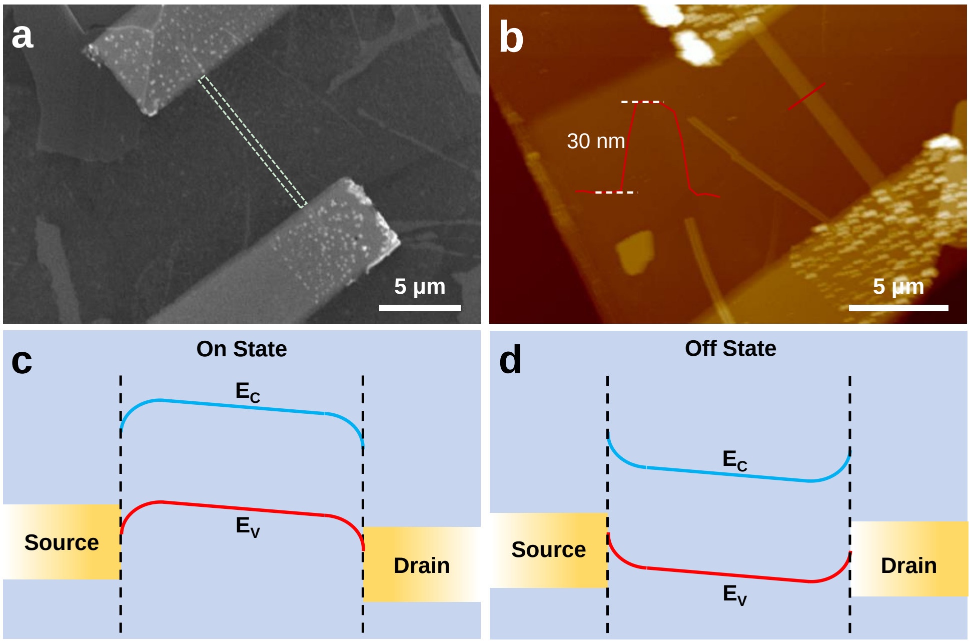

Fig. S3 Detailed morphologies of Te FET with global bottom-gate structure and the energy band diagram. (**a**) SEM image of a typical global bottom-gate FET based on Te nanobelt on h-BN/SiO_2/Si, the channel Te is indicated by the white dotted rectangular box. (**b**) AFM image of the same global bottom-gate Te FET, the inset shows the height profile. (**c, d**) The schematic energy band diagram of the Te FET contacted by Au electrodes. (**c**) On state when a negative gate voltage is applied, (**d**) off state when a positive gate voltage is applied

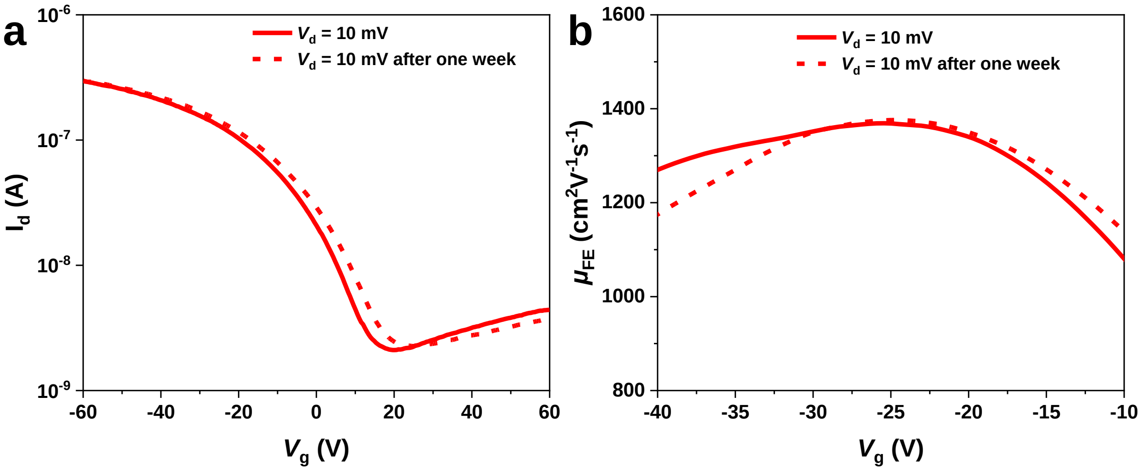

Fig. S4 Stability of Te FET with global bottom-gate structure. (**a**) Transfer curves of a typical FET based on Te on h-BN substrate measured at once and after one week, respectively. (**b**) Field-effect mobilities extracted from panel (**a**)

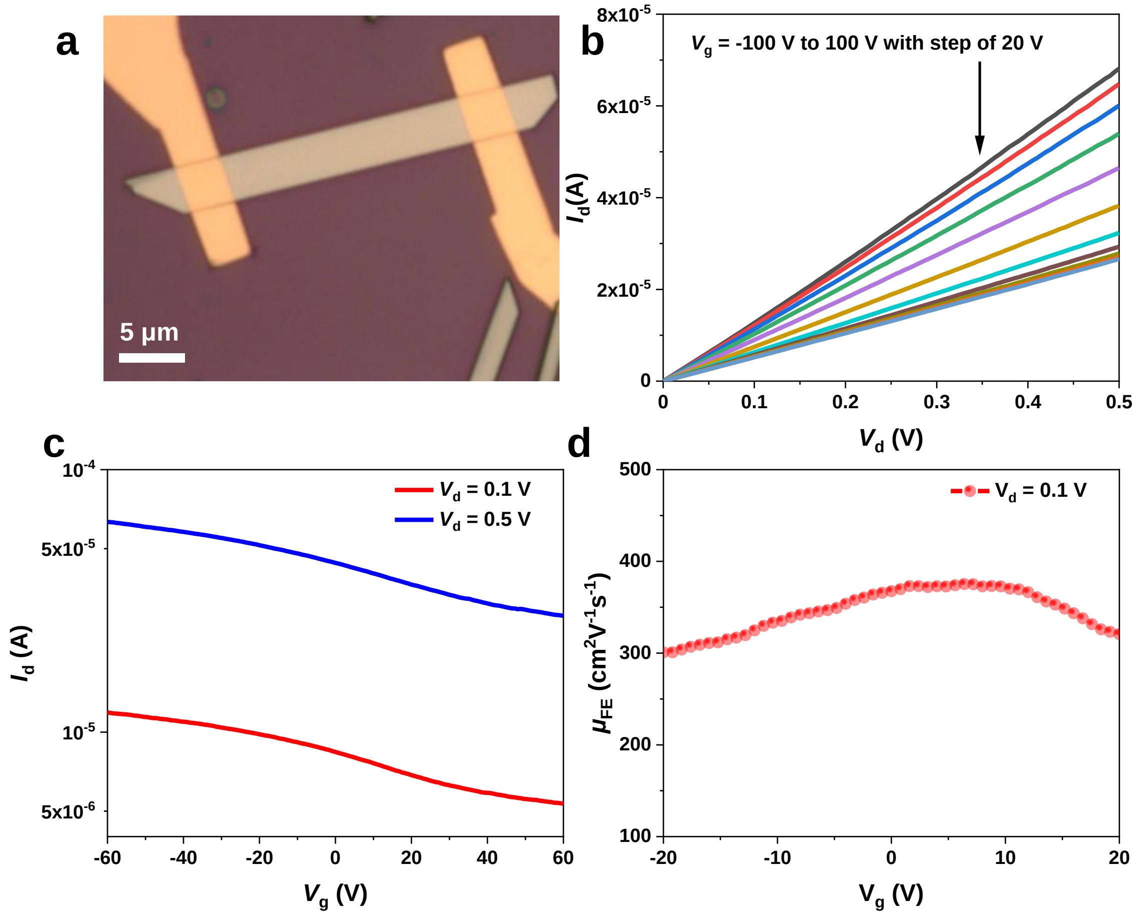

Fig. S5 Electrical performance of Te FET on bare SiO_2/Si. (**a**) Optical image of a typical Te FET with global bottom-gate structure on SiO_2/Si. Output (**b**) and transfer (**c**) curves of the Te FET in (**a**) measured at room temperature. (**d**) Field-effect mobility of Te FET extracted from the transfer curves under the bias of V_d = 0.1 V in panel (**c**)

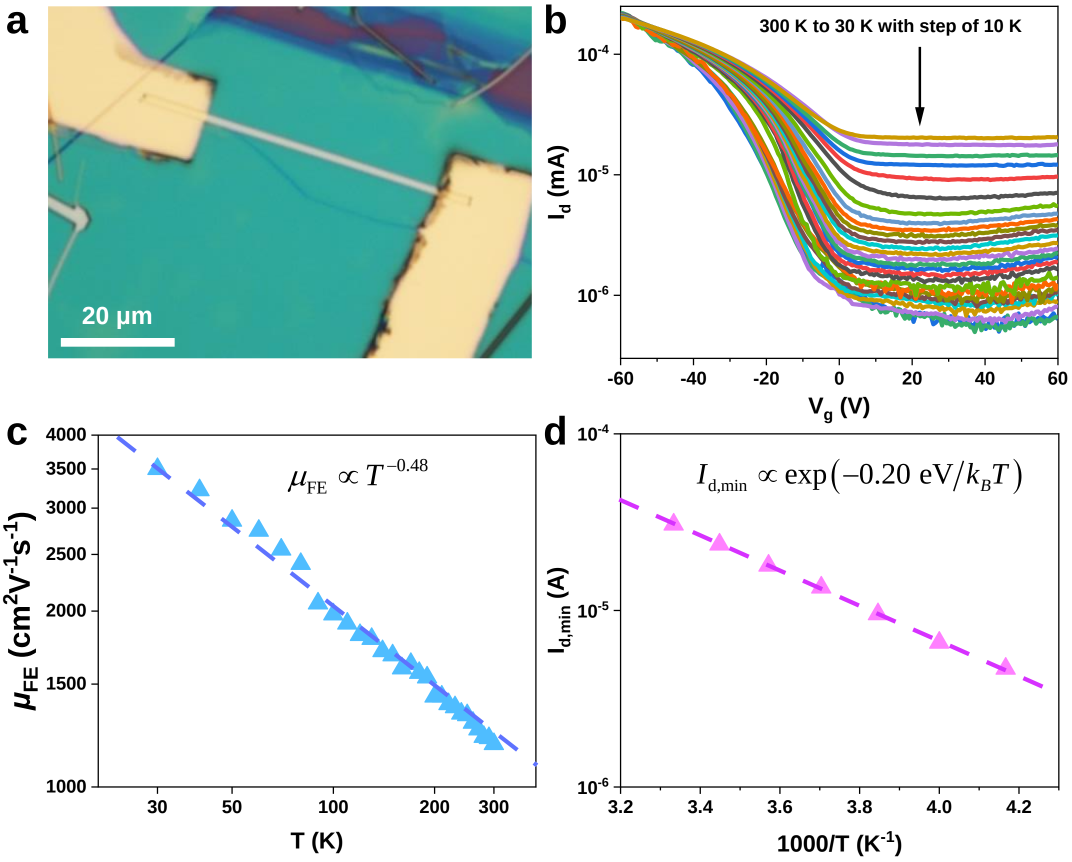

Fig. S6 Temperature-dependent electronic transport properties. (**a**) Optical image of a typical Te FET on h-BN/SiO_2/Si. (**b**) Temperature-dependent transfer curves of Te FET in (**a**) under the same bias voltage of V_{ds} = 10 mV. (**c**) Field-effect mobility in our Te FET as a function of temperature and (**d**) Arrhenius plot showing the temperature dependent minimum drain source current ($I_{d,min}$) as a function extracted in panel (**b**)

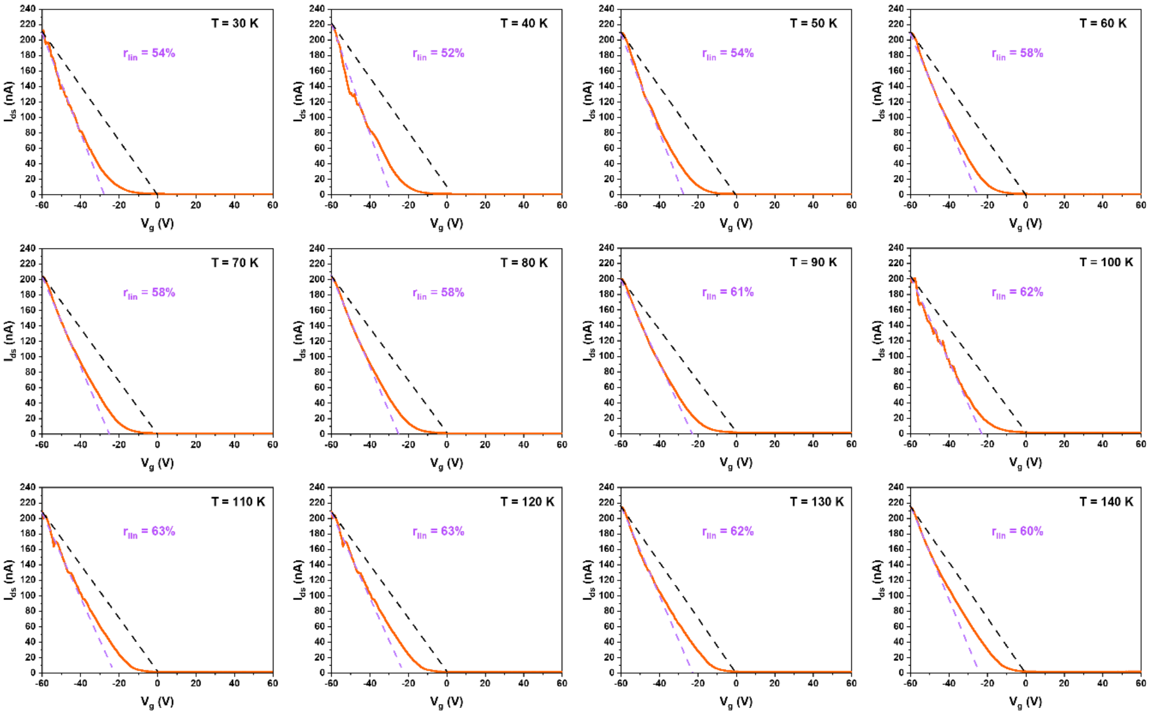

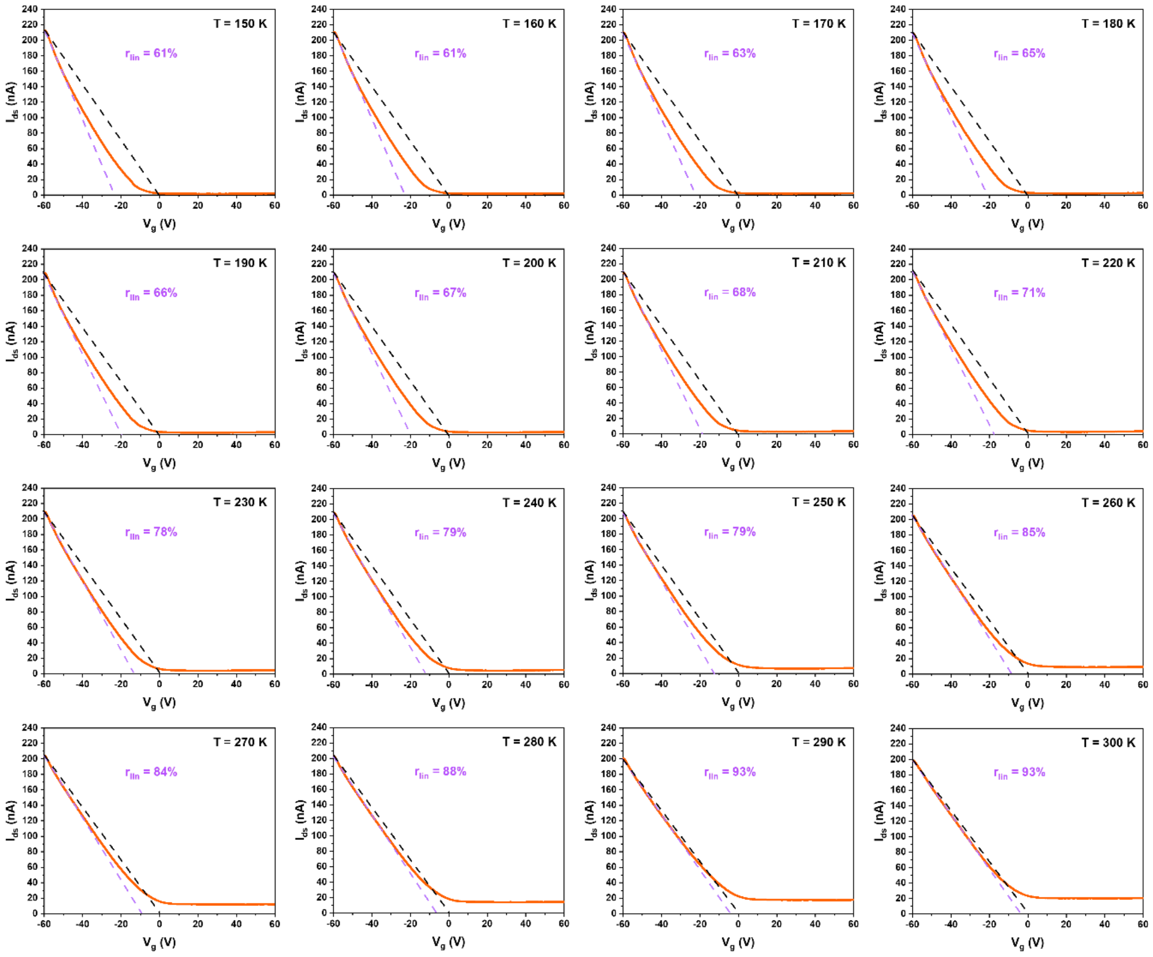

Fig. S7 Temperature-dependent transfer curves under the bias voltage of V_{ds} = 10 mV. The black and pink dashed lines in each panel represent the slope of an electrically equivalent FET and the linear regime used to extract the claimed mobility, respectively

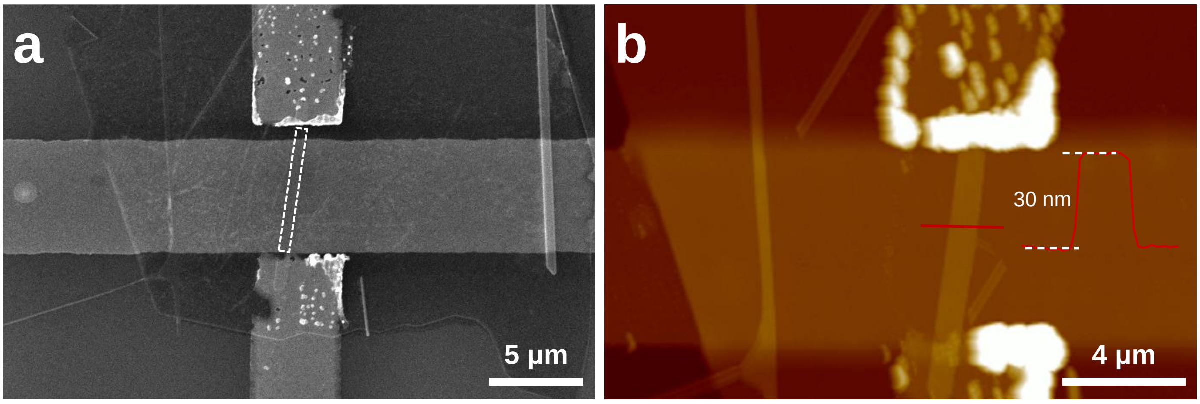

Fig. S8 Detailed morphologies of Te FET with local-bottom gate structure. (**a**) SEM image of a local bottom-gate FET based on Te nanobelt on h-BN, the channel Te is indicated by the white dotted rectangular box. (**b**) AFM image of the same local bottom-gate Te FET, the inset shows the height profile

Table S1 A detailed comparison of Te-based FET with other reported vdW semiconductor-based FET

vdW materials	Field-effect mobility ($cm^2V^{-1}s^{-1}$)	On/off ratio	References
Te	1370	10^2	**This work**
	700	500	[23]
	450	2×10^3	[32]
	100 - 500	< 10	[33]
	2.3	< 10	[37]
bP	~1000	10^5	[17]
WSe_2	140	$>10^7$	[42]
$PtSe_2$	210	10^2	[13]
MoS_2	167 ± 20	10^6	[45]
ReS_2	2.6	10^7	[47]

Table S2 Statistic of field-effect mobility and on/off ratio of the Te FETs on h-BN/SiO_2/Si substrate

Device number	Field-effect mobility (cm2V-1s-1)	On/off ratio
1	1240	10
2	1015	34
3	950	22
4	1300	12
5	1045	110
6	1262	8
7	847	8
8	923	20
9	1300	15
10	1200	5

Table S3 Statistic of field-effect mobility and on/off ratio of the investigated Te FETs on bare SiO_2/Si substrate

Device number	Field-effect mobility (cm2V-1s-1)	On/off ratio
1	525	2
2	306	1.3
3	370	14
4	375	3
